# Toward Large‐Scale Photonic Chips Using Low‐Anisotropy Thin‐Film Lithium‐Tantalate

**DOI:** 10.1002/advs.202410345

**Published:** 2025-01-10

**Authors:** Fei Huang, Xiaowan Shen, Siyuan Wang, Haochen Xu, Hongxuan Liu, Zexu Wang, He Gao, Xinmin Yao, Hengzhen Cao, Bin Chen, Xijie Wang, Jizhi Zhang, Zhile Wu, Mingyu Zhu, Hongzhi Xiong, Weike Zhao, Huan Li, Zejie Yu, Liu Liu, Yaocheng Shi, Daoxin Dai

**Affiliations:** ^1^ College of Optical Science and Engineering Zhejiang University Hangzhou 310058 China; ^2^ ZJU‐Hangzhou Global Scientific and Technological Innovation Center Zhejiang University Hangzhou 311215 China; ^3^ State Key Laboratory for Extreme Photonics and Instrumentation College of Optical Science and Engineering International Research Center for Advanced Photonics Zhejiang University Hangzhou 310058 China; ^4^ Ningbo Research Institute Zhejiang University Ningbo 315100 China

**Keywords:** electro‐optic modulation, lithium tantalate, optical transmitter, photonics chips, wavelength division multiplex

## Abstract

Photonic manipulation of large‐capacity data with the advantages of high speed and low power consumption is a promising solution for explosive growth demands in the era of post‐Moore. A well‐developed lithium‐niobate‐on‐insulator (LNOI) platform has been widely explored for high‐performance electro‐optic (EO) modulators to bridge electrical and optical signals. However, the photonic waveguides on the x‐cut LNOI platform suffer serious polarization‐mode conversion/coupling issues because of strong birefringence, making it hard to realize large‐scale integration. Here, low‐birefringence photonic integrated circuits (PICs) based on lithium‐tantalate‐on‐insulator (LTOI) are proposed and demonstrated, which enables high‐performance passive photonic devices as well as EO modulators, showing great potential for large‐scale photonic chips. Analysis of mode conversion and evolution behaviors with both low‐ and high‐birefringence shows undesired mode hybridizations can be effectively suppressed. A simple and universal fabrication process is developed and various representative passive photonic devices are demonstrated with impressive performances. Finally, a wavelength‐division‐multiplexed optical transmitter is developed with a data rate of 1.6 Tbps by monolithically integrating 8 EO modulators and an 8‐channel arrayed waveguide grating. Therefore, the demonstrated low‐birefringence LTOI platform shows strong ability in both passively and actively controlling photon behaviors on a chip, indicating great potential for ultrafast processing and communicating large‐capacity data.

## Graphical abstract

This article demonstrates low‐birefringence photonic integrated circuits based on lithium‐tantalate‐on‐insulator, enabling high‐performance passive photonic devices as well as electro‐optic (EO) modulators for large‐scale photonic chips. Various representative passive photonic devices are demonstrated with impressive performances. An optical transmitter with a data rate of 1.6 Tbps is further realized by monolithically integrating 8 EO modulators and an 8‐channel arrayed waveguide grating.

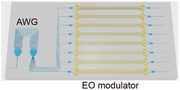

## 1 Introduction

The emergence of big data and artificial intelligence has demanded explosive growth of data processing and communication capacity in recent years.^[^ ^1^, ^2^, ^3^, ^4^, ^5^, ^6^ ^]^ Electronic integrated circuits gradually fail to satisfy the growing data capacity requirement due to the well‐known reason for “the end of Moore's law.” Photonic integrated circuits (PICs) with the advantages of large bandwidth and low power consumption are recognized as promising candidates in the post‐Moore era for efficiently manipulating massive amounts of data. Electro‐optic (EO) modulators bridging the connections between electrical and optical signals play a crucial role in PICs. Thin‐film lithium niobate photonics with not only a large EO coefficient but also overall excellent optical properties such as low loss and wide transparent window have attracted extensive attention. In recent years, numerous high‐performance EO modulators^[^ ^7^, ^8^, ^9^, ^10^, ^11^, ^12^, ^13^, ^14^, ^15^, ^16^, ^17^ ^]^ and related large‐capacity data manipulation^[^ ^18^, ^19^, ^20^, ^21^, ^22^, ^23^ ^]^ have been realized. The performance of a Mach‐Zehnder interferometer (MZI) EO modulator has been pushed to 3‐dB bandwidth beyond 100 GHz under CMOS driving voltage.^[^ ^24^ ^]^ A coherent in‐phase and quadrature EO modulator has achieved a data rate of 1.96 Tbit s^−1^ for a single wavelength with the assistance of advanced digital signal processing.^[^ ^25^ ^]^

Materials with EO coefficients usually have birefringence because EO interactions are aroused from non‐centrosymmetric crystal structures. Currently, the majority of demonstrated high‐performance EO modulators are through modulating TE modes on *x*‐cut wafers,^[^ ^7^, ^8^, ^9^, ^10^, ^11^, ^12^, ^13^, ^14^, ^15^, ^16^, ^17^ ^]^ so the light of TE modes will have different effective refractive indices in different crystal directions. Relatively large birefringence of lithium niobate (*n_o_* = 2.2111 and *n_e_* = 2.1376@1550 nm) causes many undesired mode conversions and hybridizations when light travels through bent or taper waveguides.^[^ ^26^, ^27^, ^28^ ^]^ Moreover, the variation of the refractive index in different crystal directions makes it hard to define fixed optical distance and phase devices with complex layouts such as arrayed waveguide gratings and orbital angular momentum generators. As a result, the birefringence of LN severely restricts the realization of high‐performance and multifunctional passive devices, becoming the bottleneck in developing large‐scale lithium niobate PICs to handle large‐capacity data transmissions. Utilizing TM‐polarization modes on *z*‐cut LN wafers might be an option to solve the birefringence problem because the in‐plane refractive index remains identical in different crystal directions and TM‐polarization modes can be used to align with the crystal orientation of the maximum EO coefficient. However, the high dielectric constant of LN (ε_LN_ = 28) in the RF spectrum reduces the modulation efficiency because a silica upper cladding (ε_SiO2_ = 3.8) with a low dielectric constant should be introduced between metal and LN to minimize the optical absorption from metal. This requirement results in a significant voltage drop across the silica layer, which ultimately reduces the modulation efficiency within the LN core region. Therefore, utilizing TE modes on *x*‐cut LN wafers is a preferred choice to explore high‐performance EO modulators, but birefringence‐induced challenges in developing passive photonic devices make it hard to realize large‐scale photonic integration.

Another kind of low‐cost and commercialized ferroelectric material lithium tantalate (LT) is usually considered as an alternative to LN in many applications.^[^ ^29^, ^30^, ^31^, ^32^, ^33^, ^34^ ^]^ It has a similar EO coefficients *r_33_* ≈30 pm V^−1^, a broader transparency range of 280 nm–5.5 µm, a higher damage threshold of 240 MW cm^−2^, and an order of magnitude smaller birefringence (*n_o_* = 2.119 and *n_e_* = 2.123 @ 1550 nm) than LN.^[^ ^35^, ^36^ ^]^ Here, we propose and demonstrate low‐birefringence PICs on an LT on insulator (LTOI) platform to realize large‐scale integration for large‐capacity data manipulation. At first, mode conversion and evolution behaviors in both low‐ and high‐birefringence waveguides were analyzed and undesired mode hybridization can be effectively suppressed. Next, a simple and universal fabrication process was developed and pivotal devices including a high‐quality microring resonator with loaded *Q* of ≈1.23 × 10^6^, a compact microring resonator with free spectrum range (FSR) of ≈8.6 nm, a grating coupler with an efficiency of 4.6 dB facet^−1^, an MZI EO modulator with 3‐dB bandwidth of >67 GHz were experimentally realized. To show the advantages of low birefringence of LTOI, an 8‐channel arrayed waveguide grating (AWG) for dense wavelength division and multiplexing was demonstrated with insertion loss <3.5 dB and crosstalk <−23 dB. Further, an optical transmitter with a monolithic integration of 8 EO modulators and an AWG achieves a data rate of 1.6 Tbps. Therefore, the demonstrated LTOI PICs will open a new avenue for large‐scale integration of an EO material platform and find many applications in ultrafast signal processing and data communication.

## 2 Results

### 2.1 Mode Evolution Behaviors

**Figure 1:**
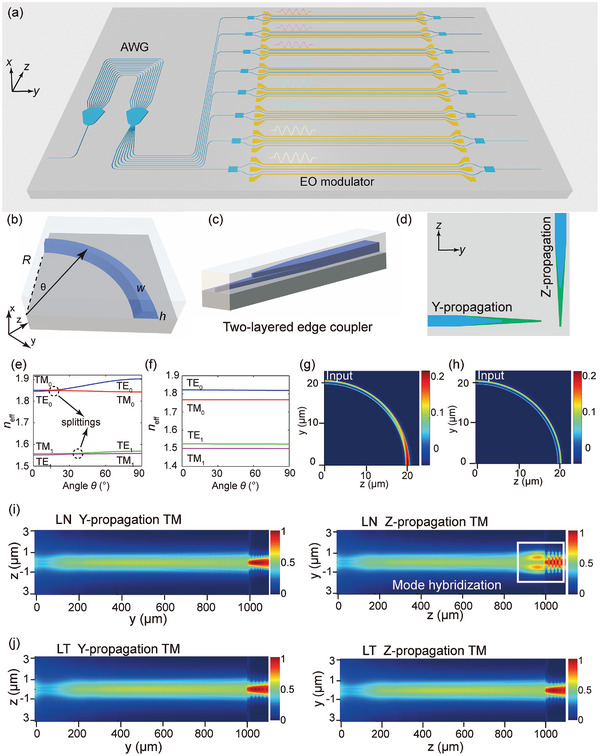
Mode evolution behaviors of LN and LT PICs. a) A schematic illustration of large‐scale integrated LT PICs. b) A schematic illustration of a bent waveguide on LNOI or LTOI platform, where R and *θ* denote the radius of the bent waveguide and the angle between the waveguide cross section and *y*‐axis. c) A schematic illustration of a two‐layered inverse taper. d) A schematic illustration of Y‐ and Z‐propagation inverse‐tapers. e,f) Effective refractive index variations of TE and TM modes as a function of propagation direction *θ* at e) LN (e) and LT (f) waveguides. g,h) Mode evolution profiles | ***E_x_*** | with the TE_0_ mode inputs along the LN (g) and LT (h) bent waveguides with a radius of 20 µm. i,j) Modal evolution profiles along Y‐ (left) and Z‐ (right) propagation 400‐nm LN (i) and 400‐nm LT (j) two‐layered inverse tapers.

**Figure** **1**a shows the schematic illustration of LT PICs, where both passive devices and high‐speed EO modulators are monolithically integrated on the chip. Low birefringence LT ensures different kinds of passive devices because of the certain phase and optical distance in different propagation directions. Fundamental optical components including a bent waveguide (Figure 1b) and a two‐layered inverse taper (Figure 1c) propagating in different directions (Figure 1d) were taken as two examples to analyze mode evolution behaviors in both LN and LT waveguides. For both LN and LT waveguides with thickness *h* = 600 nm and width *w*_1_ = 600 nm, Figure 1e,f shows the effective refractive indices of different kinds of modes as a function of propagation direction *θ* of both LN (1e) and LT (1f) waveguides, respectively. The effective refractive index variations of LN waveguides contain several splitting where two modes have non‐zero mode overlap and their effective refractive indices will cross at certain crystal directions, so undesired modal hybridizations will occur. However, effective refractive index splitting does not appear in LT waveguides, indicating these modes will not interfere with each other at bent waveguides. More effective refractive index analysis as a function of propagation direction *θ* about different orders of modes at different kinds of LN and LT waveguides are shown in Figure S1 (Supporting Information), all undesired effective refractive index crossings of LN waveguides can be avoided in LT waveguides.

As for LN and LT bent waveguides with a radius of *R* = 20 µm, Figure 1g,h shows the evolution profiles of | ***E_x_*** | component under the input of the TE_0_ mode, where the major components of the electric field are in *y‐z* planes. It indicates a large amount of | ***E_x_*** | component which belongs to TM polarization is excited in the LN bent waveguide (Figure 1g) but the | ***E_x_*** | component changes negligibly along the LT bent waveguide (Figure 1h). Moreover, a two‐layered inverse taper (Figure 1c) working as a standard model for an efficient fiber‐waveguide coupling is also restricted in LN. Figure 1i shows the modal evolution profiles along Y‐ and Z‐propagation inverse tapers under the input of the TM_0_ mode, indicating the TM_0_ mode transmits the Y‐propagation inverse taper smoothly but severe mode hybridization between the TM_0_ and TE_1_ modes along the Z‐propagation inverse taper. The reason for it is that the birefringence of the LN waveguide makes the Y‐ and Z‐propagation TE_1_ modes with dominant electric fields of ***E_z_*** and ***E_y_*** components have different effective refractive indices. The mode evolution profiles for Y‐ and Z‐propagation LT inverse‐tapers under input of the TM_0_ mode are shown in Figure 1j, indicating modal hybridization does not appear in both propagation directions. Therefore, LT PICs can avoid many undesired mode hybridizations that LN PICs face now, ensuring large‐scale integration of complex and functional PICs.

### 2.2 Passive Optical Devices

**Figure 2:**
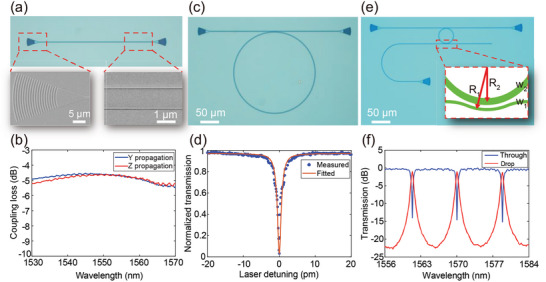
Fabricated fundamental optical components and measured results. a) Microscope image of a pair of grating couplers connected with a straight waveguide. The insets show the SEM images zoomed in at the grating coupler and waveguide. b) Measured transmission of Y‐ (blue) and Z‐propagation (red) grating couplers. c) Optical microscope image of the fabricated microring resonator with a radius of 108 µm. d) Measured (dots) and fitted (line) transmission. e) Optical microscope image of the fabricated add‐drop microring resonator with a radius of 20 µm. f) Measured transmission of the drop (red) and through (blue) ports.

After careful analysis of mode evolution behaviors in high‐birefringence LN and low‐birefringence LT waveguides, we designed and demonstrated pivotal devices that are of fundamental importance to large‐scale LT PICs on a 400‐nm LTOI platform. **Figure** **2**a shows the optical microscope image of a pair of focusing grating couplers connected by a straight waveguide, where the two insets are scanning electron microscope (SEM) images zoomed in at the grating coupler and waveguide. Figure 2b shows the measured transmission spectra of Y‐ and Z‐propagation grating couplers with identical structural parameters of a period of 0.99 µm and duty cycle of 40%, meriting the similar performance of minimum coupling loss of 4.6 dB and 1‐dB bandwidth over 40 nm. Figure 2c shows the optical microscope image of the fabricated microring resonator with a radius of 108 µm, waveguide width of 1.5 µm, and the gap between bus and microring waveguides of 0.5 µm. The measured (dots) and fitted (line) transmission spectra plotted in Figure 2d indicate that loaded and intrinsic quality factors can reach 1.23 × 10^6^ and 2.09 × 10^6^, respectively, corresponding propagation loss is only ≈0.2 dB cm^−1^. Figure 2e shows the optical microscope image of the fabricated add‐drop microring resonator with a radius of only ≈20 µm, the coupling between bus and microring waveguides is designed with phase matching *R*_1_ *n*_eff1_ = *R*_2_ *n*_eff2_ as shown in the inset of Figure 2e to enhance the coupling efficiency. Low‐birefringence of the LT waveguide suppresses the mode hybridization at a small bent waveguide so low insertion loss of 1.0 dB, high extinction ratio of 21.5 dB, and large FSR of 8.6 nm are realized simultaneously as plotted in Figure 2f.

**Figure 3:**
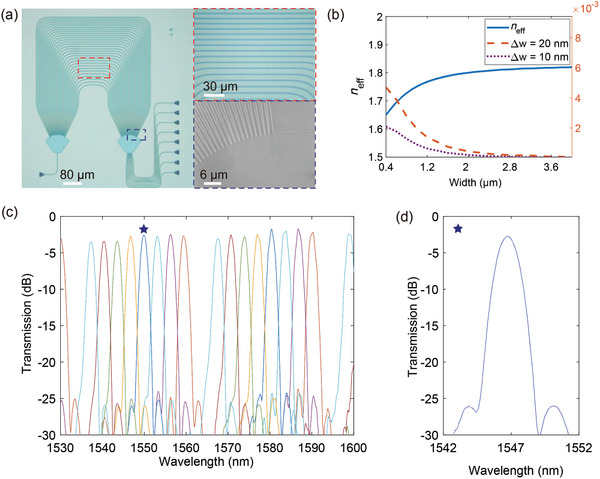
Design and measurement of an AWG. a) Optical microscope image of the fabricated devices, where the insets are zoomed in at arrayed waveguide and FPR regions. b) Effective refractive index (solid) and refractive index changes with width variations of 10 nm (dotted) and 20 nm (dashed) as functions of waveguide width. The left y‐axis corresponds to the solid curve. The right y‐axis is shared by the dotted and dashed curves. c) Measured transmission of the fabricated AWG. d) A typical transmission of a single channel, which is marked with a star at c.

To show the advantages of low birefringence of LT, we demonstrated an AWG with a strict requirement on phase certainty in different crystal directions on an *x*‐cut LTOI platform. **Figure** **3**a shows the optical microscope image of a fabricated 8‐channel AWG, which has a central input port and 8 output ports. Several special designs (see in the Experimental Section) were considered in the demonstrated devices to improve the performances such as insertion loss and crosstalk. In order to overcome the phase error problems caused by imperfect fabrication, the arrayed waveguides are designed with a width of 2 µm, which is far beyond the single mode regime, to reduce the influence of waveguide width uncertainly. Figure 3b plots the simulated effective refractive index (solid) as a function of waveguide width, indicating the effective refractive index of the TE_0_ mode gradually becomes flattened with the increase of waveguide width. The effective refractive index changes with waveguide width variations of 10 nm (dotted) and 20 nm (dashed) as a function of waveguide width presented in Figure 3b also proves that the effective refractive index of a wider waveguide is less sensitive to waveguide width, contributing to reducing phase errors of arrayed waveguides under the same fabrication condition. In order to measure the spectra of the fabricated AWG. Figure 3c shows the measured transmission spectra of all 8 channels normalized with respect to the transmission of a straight waveguide with identical grating couplers, indicating the 8‐channel AWG has channel spacings of 3.2 nm, insertion losses <3.5 dB, crosstalk between the adjacent channels <−23 dB. The AWG on the LTOI platform demonstrates excellent insertion loss and crosstalk performance. The zoomed‐in transmission of a single channel plotted in Figure 3d conveys each channel has a 3‐dB bandwidth of 1.54 nm. Moreover, we demonstrated AWGs along with different crystal directions on both 400‐nm and 600‐nm LTOI platforms to prove the universality of the advantages of low‐birefringence. The measured results all show these AWGs have low insertion loss and low crosstalk as shown in Figure S2 (Supporting Information).

### 2.3 High‐Speed EO Modulation and Large‐Capacity Data Transmission

**Figure 4:**
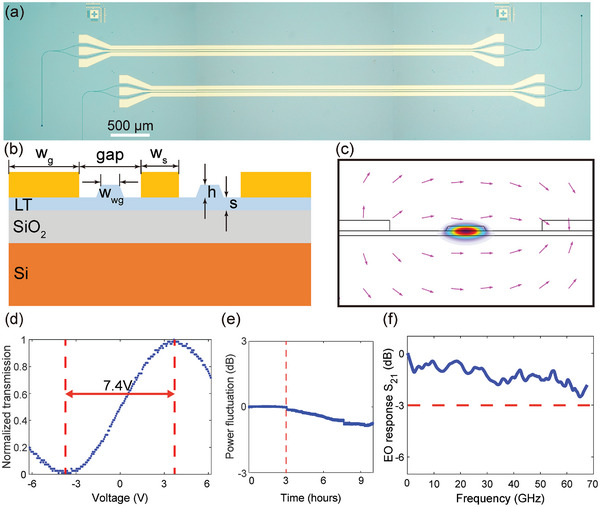
Integrated high‐speed LT EO modulators. a) Optical microscope image of the fabricated EO modulator. b) Schematic cross‐sectional view of the proposed EO modulator. c) Simulated cross‐sectional view of electric and optical field distribution of the proposed EO modulator. d) Measured optical transmission as a function of driving voltage. e) Measured power fluctuation from an LTOI MZI EO modulator biased at quadrature. f) Measured small signal response with a 3‐dB bandwidth beyond 67 GHz.

We turned our attention to EO modulation performance on the LTOI platform after demonstrating high‐performance passive devices resulting from low birefringence. An integrated LT EO modulator was demonstrated with a modulation efficiency of V_π_L = 3.7 V·cm and a 3‐dB EO modulation bandwidth >67 GHz. **Figure** **4**a shows the optical microscope image of the fabricated MZI EO modulators on LTOI platform, composed of two 3‐dB multimode interference couplers that split and combine the optical power, along with a 5‐mm push‐pull optical waveguide phase shifter pair. The phase shifter pair supporting confined co‐propagation microwave and optical fields are required to have matched group velocities and low propagation losses. Figure 4b shows the schematic cross‐section of the EO modulator, where electrodes are arranged in a ground‐signal‐ground (GSG) configuration, with optical waveguides traversing through the gaps of a GSG coplanar microwave strip line. The microwave and optical group velocities on LTOI platform are designed independently because the light field is limited to the waveguide region and the microwave mode mainly exists in the substrate. As a result, the device can maintain a speed match between optical and microwave signals at a very high microwave frequency without compromising electro‐optical efficiency. The waveguide width *w_wg_* of 1.5 µm, electrode thickness of 450 nm, signal electrode width *w_s_* of 18 µm, ground electrode width *w_g_* of 50 µm, and electrode gap of 6.5 µm were obtained after careful optimization using the finite element method, achieving microwave and optical group refractive index matching of ≈2.23, traveling wave electrode impedance ≈50 Ω, and neglectable optical absorption loss from metal simultaneously. Figure 4c shows the simulated electrical and optical field distributions in one phase shifter of the optimized EO modulator. The static EO response was measured with a 1‐kHz triangular voltage sweep method. The measured results plotted in Figure 4d indicate half wave voltage V_π_ for the 5‐mm EO modulator is 7.4 V and the corresponding V_π_·L is 3.7 V·cm. In addition, we recorded the output power fluctuation as a function of time for the EO modulation biased at quadrature. The measured result indicates a power fluctuation of less than 0.1 dB over a 3 h duration, as shown in Figure 4e, which is significantly lower than the static drift observed in LNOI MZI EO modulator.^[^ ^11^ ^]^ Figure 4f shows the measured bandwidth of the small‐signal EO response (S_21_) of the fabricated EO modulator, indicating 3‐dB bandwidth of the fabricated EO modulator is beyond 67 GHz. It should be noted that the presently measured modulation bandwidth is limited by the experimental setup. The EO bandwidth of a modulator is mainly limited by the loss of the traveling‐wave electrodes in the case of the velocity matching. We believe that the EO bandwidth of the modulator could be extended beyond 100 GHz with more advanced characterization equipment.

**Figure 5:**
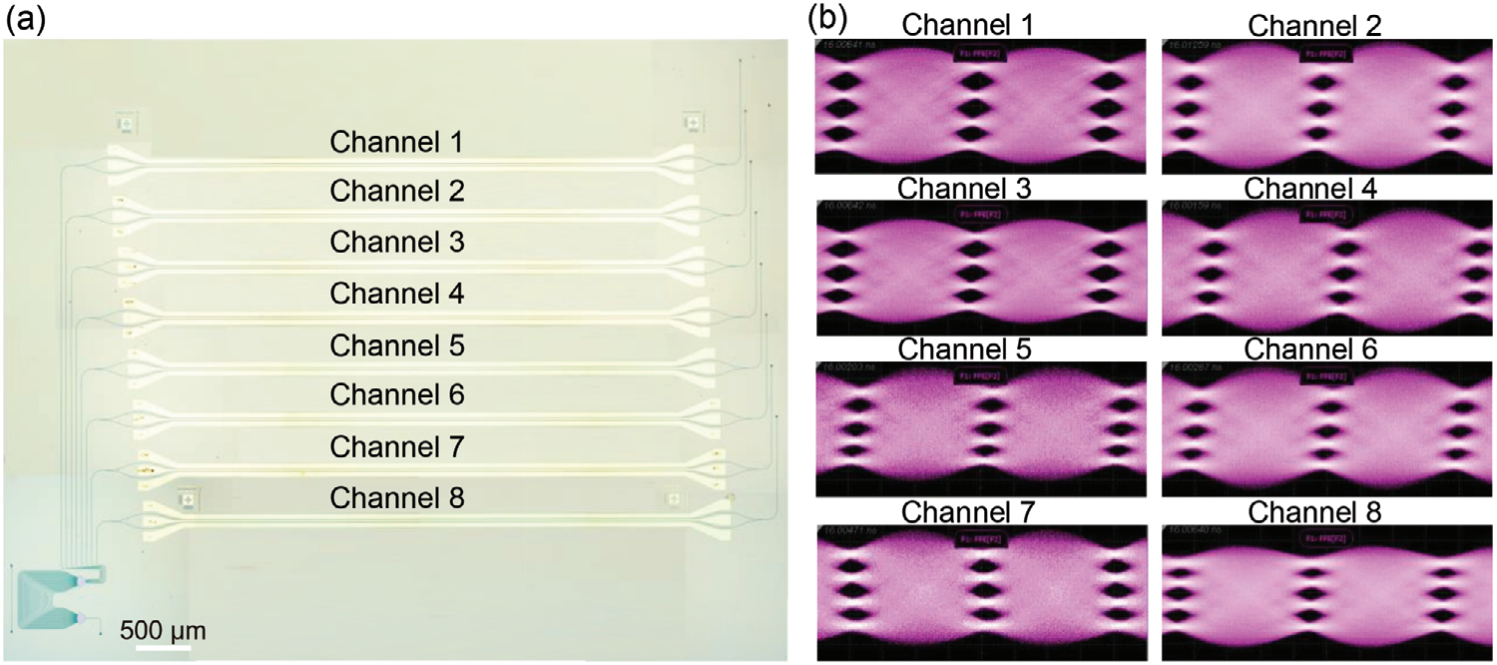
An integrated large‐capacity transmitter. a) Optical microscope image of the fabricated 8‐channel transmitter. b) Measured 100 GBaud eye diagrams of all channels.

A large‐capacity optical transmitter was demonstrated by monolithically integrating an MZI EO modulator array and an 8‐channel AWG, as shown in **Figure** **5**a, enabling sending data streams at 8 wavelengths to a single waveguide to significantly enhance the link capacity. The 3‐dB EO modulation bandwidth of each EO modulator was characterized at first, and all of them show 3‐dB modulation bandwidth beyond 67 GHz as shown in Figure S7 (Supporting Information). High‐speed data generation using the fabricated optical transmitter was characterized experimentally. Figure 5b shows the measured eye diagrams of 4‐level pulse amplitude modulation (PAM4) signals at the data rates of 100 GBaud, indicating eye diagrams of all channels are well open. As a result, the demonstrated optical transmitter can achieve a data capacity of at least 1.6 Tbps.

## 3 Conclusion

In this paper, we developed and demonstrated low‐birefringence PICs based on large‐volume commercially available LT. Mode conversion and evolution behaviors in both low‐ and high‐birefringence waveguides have been thoroughly analyzed and low‐birefringence has been proved to have advantages over high‐birefringence for many passive devices requiring certain optical phase, distance, and modes. AWGs with strict requirements on optical phase, distance, and modes certainly were demonstrated with channel spacing of 3.2 nm, channel number of 8, insertion loss of <3.5 dB, and crosstalk of <−23 dB for dense wavelength division and multiplexing. Further, a large‐scale integrated optical transmitter by monolithic integration of 8 EO modulators and an AWG was demonstrated with a data rate of 1.6 Tbps. Therefore, our work successfully proving the special low‐birefringence property of LT overcomes the bottlenecks of LN in exploring passive devices, making it possible to push integration scale to a level beyond LN and find more applications in data center interconnects, long‐haul optical communications, and quantum information after overcoming the challenges of ultralow loss waveguides, polarization‐insensitive devices, wafer‐scale precise fabrication, etc. Moreover, LT has many other advantages such as a high optical damage threshold, a low direct current drift, and good acoustic properties, which are highly desired for exploring high‐power nonlinear photonics applications, ultrafast optical switches, and acousto‐optic interactions.

## 4 Experimental Section

### Design of AWGs

The design of AWGs follows the method as shown in Figure S6a (Supporting Information). At first, an adiabatic taper was introduced to connect the free propagation region (FPR) and the arrayed waveguide region (AWR). The input width *w*_1_ of an adiabatic taper and gap *w*_2_ between two adiabatic tapers were designed as 2 and 0.5 µm to minimize the mode‐mismatch‐induced loss between FPR and AWR. The output width of an adiabatic taper was chosen as 0.6 µm to filter out the higher‐order modes excited in the boundary of FPR and AWR. Despite a wide waveguide could be used to reduce the phase error caused by imperfect fabrication, it also excited many undesired higher‐order modes at the junction of straight and bent waveguides. Usually, a bent waveguide with a radius large enough will be chosen to reduce the excitation of higher order modes, but the method will not only increase the footprint of devices but also deteriorate the phase errors because of the longer length of arrayed waveguides. Therefore, a 90° Euler‐bend composed of a pair of 45° modified‐Euler‐bends with gradient‐varying curvature along the propagation direction was introduced to suppress curvature mutations as shown in Figure S6b (Supporting Information). The maximum radius *R_max_* was set as 1000 µm to make modal field distributions in the bent waveguide very similar to those in the straight waveguide. The minimum bending radius *R_min_*, playing a critical role in achieving low losses and minimal mode crosstalk, was set as *R_min_* = 40 µm to balance footprint, crosstalk, and loss. The 3D‐FDTD solver module was used for simulating the light propagation and achieving the spectral analysis in theory. Figure S6c (Supporting Information) shows the simulated light propagation when the TE_0_ mode was launched along the designed Euler‐bend. It could be observed that the light propagation process was exceptionally smooth without the interference‐induced ripples. With this design, the excess loss of the TE_0_ mode is <0.01 dB in the wavelength range of 1500–1600 nm, while the inter‐mode crosstalk is <−30 dB as shown in Figure S6d (Supporting Information).

### Fabrication

Devices were fabricated on a 400‐nm *x*‐cut LT on a 3‐µm silica with a 525‐µm‐thick Si handle substrate. All passive devices were first patterned by a step of electron‐beam lithography (EBL), and then the pattern was transferred into the LT layer by Ar^+^ plasma dry etch, in which way the etch depth was 200 nm and the etched waveguide had a sidewall angle α ≈60°. The next step of UV lithography was used to form patterns of electrodes for EO modulation devices, a layer of 10/450‐nm Ti/Au electrodes was deposited by steps of electron‐beam evaporation and lift‐off.

### Metrology*—*Grating Couples and AWGs

Light from a broadband amplified spontaneous emission source was collected by an optical spectrum analyzer (OSA) after transmitting through the devices under test via a pair of grating couplers.

### Metrology—Microring Resonators

The limited resolution of an OSA could not characterize the high *Q* properties, so the measurement process followed light from a tunable semiconductor laser with the polarization controlled by a polarization controller was sent into the device under test via a grating coupler, then light coupled out of the device under test was collected by a photodetector.

### Metrology—3‐dB Modulation Bandwidth of EO Modulators

The experimental setup characterizing small signal response is depicted in Figure S7a (Supporting Information). A 1550‐nm laser with polarization adjusted by a polarization controller was coupled into the waveguide through the grating coupler after passing by a variable optical attenuator and an erbium‐doped fiber amplifier. The radio frequency (RF) signal was applied to the TWEs through a 67‐GHz RF probe and the RF signal after transmitting to the device was terminated by attaching a second RF probe connected with a 50 Ω terminator. The modulated light was coupled out of the chip through another grating coupler and then collected and analyzed by a 67‐GHz light component analyzer.

### Metrology—Eye diagrams

The experimental setup characterizing eye diagrams is depicted in Figure S7b (Supporting Information). The electrical signal generated by a clock source was transformed into the PAM4 signal by an arbitrary waveform generator. The PAM4 signal combined with static voltage using a bias‐tee was then applied to the fabricated device, and the modulated optical signal was collected by a photodetector and subsequently connected to a real‐time oscilloscope.

## Conflict of Interest

The authors declare no conflicts of interest.

## Supporting information



Supporting Information

## Data Availability

The data that support the findings of this study are available from the corresponding author upon reasonable request.
