# Supplementary material for: Toward Large‐Scale Photonic Chips Using Low‐Anisotropy Thin‐Film Lithium‐Tantalate
Source: Adv Sci (Weinh). 2025 Jan 10;12(9):2410345. doi: 10.1002/advs.202410345 (PMC11884565; doi:10.1002/advs.202410345)
Supplement: Supplementary file 1 — Supporting Information [file ADVS-12-2410345-s001.docx]

**Supporting Information for Towards large-scale photonic chips using low-anisotropy thin-film lithium-tantalate**

*Fei Huang, Xiaowan Shen, Siyuan Wang, Haochen Xu, Hongxuan Liu, Zexu Wang, He Gao, Xinmin Yao, Hengzhen Cao, Bin Chen, Xijie Wang, Jizhi Zhang, Zhile Wu, Mingyu Zhu, Hongzhi Xiong, Weike Zhao, Huan Li, Zejie Yu***, Liu Liu, Yaocheng Shi and Daoxin Dai*

F. Huang, X. Shen, S. Wang, H. Xu, H. Liu, Z. Wang, H. Gao, X. Yao, H. Cao, B. Chen, X. Wang, J. Zhang, Z. Wu, M. Zhu, H. Xiong, W. Zhao, H. Li, Z. Yu, L. Liu, Y. Shi, D. Dai

College of Optical Science and Engineering, Zhejiang University, Hangzhou 310058, China

H. Li, Z. Yu, L. Liu, Y. Shi, D. Dai

ZJU-Hangzhou Global Scientific and Technological Innovation Center, Zhejiang University, Hangzhou 311215, China

State Key Laboratory for Extreme Photonics and Instrumentation, College of Optical Science and Engineering, International Research Center for Advanced Photonics, Zhejiang University, Hangzhou 310058, China

Y. Shi, D. Dai

Ningbo Research Institute, Zhejiang University, Ningbo 315100, China.

E-mail: zjyu@zju.edu.cn

**Figure S1.** Effective refractive indices of different kinds of modes as a function of axis directions in different kinds of LN and LT waveguides. (a) Effective refractive indices variations of TE_0_, TE_1_, TE_2_, and TM_0_ modes in a LN waveguide with thickness *t* = 400 nm, etch depth *h* = 200 nm, and waveguide width *w* = 2 µm. (b) Effective refractive indices variations of TE_0_, TE_1_, TE_2_, and TM_0_ modes in a LT waveguide with thickness *t* = 400 nm, etch depth *h* = 200 nm, and waveguide width *w* = 2 µm. (c) Effective refractive indices variations of TE_0_, TE_1_, TE_2_, and TM_0_ modes in a LN waveguide with thickness *t* = 600 nm, etch depth *h* = 300 nm, and waveguide width *w* = 2 µm. (d) Effective refractive indices variations of TE_0_, TE_1_, TE_2_, and TM_0_ modes in a LT waveguide with thickness *t* = 600 nm, etch depth *h* = 300 nm, and waveguide width *w* = 2 µm.

**Figure S2.** Experimentally measured results of different kinds of AWGs. (a) Measured transmission of the 8-channel Z-propagation AWG fabricated on a 400-nm LTOI platform with a waveguide etch depth of 200 nm. (b) A typical transmission of a single channel in (a). (c) Measured transmission of the 8-channel Y-propagation AWG fabricated on a 600-nm LTOI platform with waveguide etch depth of 300 nm. (d) A typical transmission of a single channel in (c). (e) Measured transmission of the 8-channel Z-propagation AWG fabricated on a 600-nm LTOI platform with waveguide etch depth of 300 nm. (f) A typical transmission of a single channel in (e).

We performed simulations of LNOI and LTOI AWGs using the following processes. The input FPRs of both x-cut LNOI and LTOI AWGs are modeled in 3D FDTD using Lumerical. The optical field (amplitude and phase) in each array waveguide at the output end is recorded as shown in **Figure S3**a. The output FPRs of both x-cut LNOI and LTOI AWGs are modeled in 3D FDTD using Lumerical as shown in Figure S3b. Figure S3c shows the simulated modal profiles in output FPRs of the LNOI and LTOI AWGs, the LN AWG cannot focus on the output of the FPR. **Figure 3d** plot the simulated spectral responses of the designed AWG on both LNOI and LTOI platforms, conveying the information that AWG on LNOI suffers large insertion loss and crosstalk.

**Figure S3.** (a) Simulation model of the input FPR. (b) Simulation model of the output FPR. (c) Simulated optical field distributions at the output FPR on both LNOI and LTOI platform. (d) Simulated spectral responses of the designed AWG on both LNOI and LTOI platforms.

We designed and fabricated an 8-channel AWG with channel spacings of 3.2 nm on an x-cut 400-nm LN platform. **Figure S4**a shows the optical microscope image of the fabricated device and Figure S4b shows the measured transmission spectra of all 8 channels, clearly indicating the fabricated AWG on lithium niobate almost cannot realize the function of wavelength division and multiplexing.

**Figure S4.** (a) Microscope image of the fabricated LN AWG. (b) Measured transmission of the fabricated AWG on LNOI.

**Figure S5.** Measured EO modulation bandwidth for all 8 channels of the fabricated integrated transmitter.

**Figure S6.** Design of AWGs. (a) A schematic illustration of AWG design consideration. (b) A schematic illustration of an Euler waveguide bend. (c) Mode profiles of light transmitting through an Euler waveguide bend. (d) Mode analysis at the output of the Euler waveguide bend under the TE_0_ mode input.


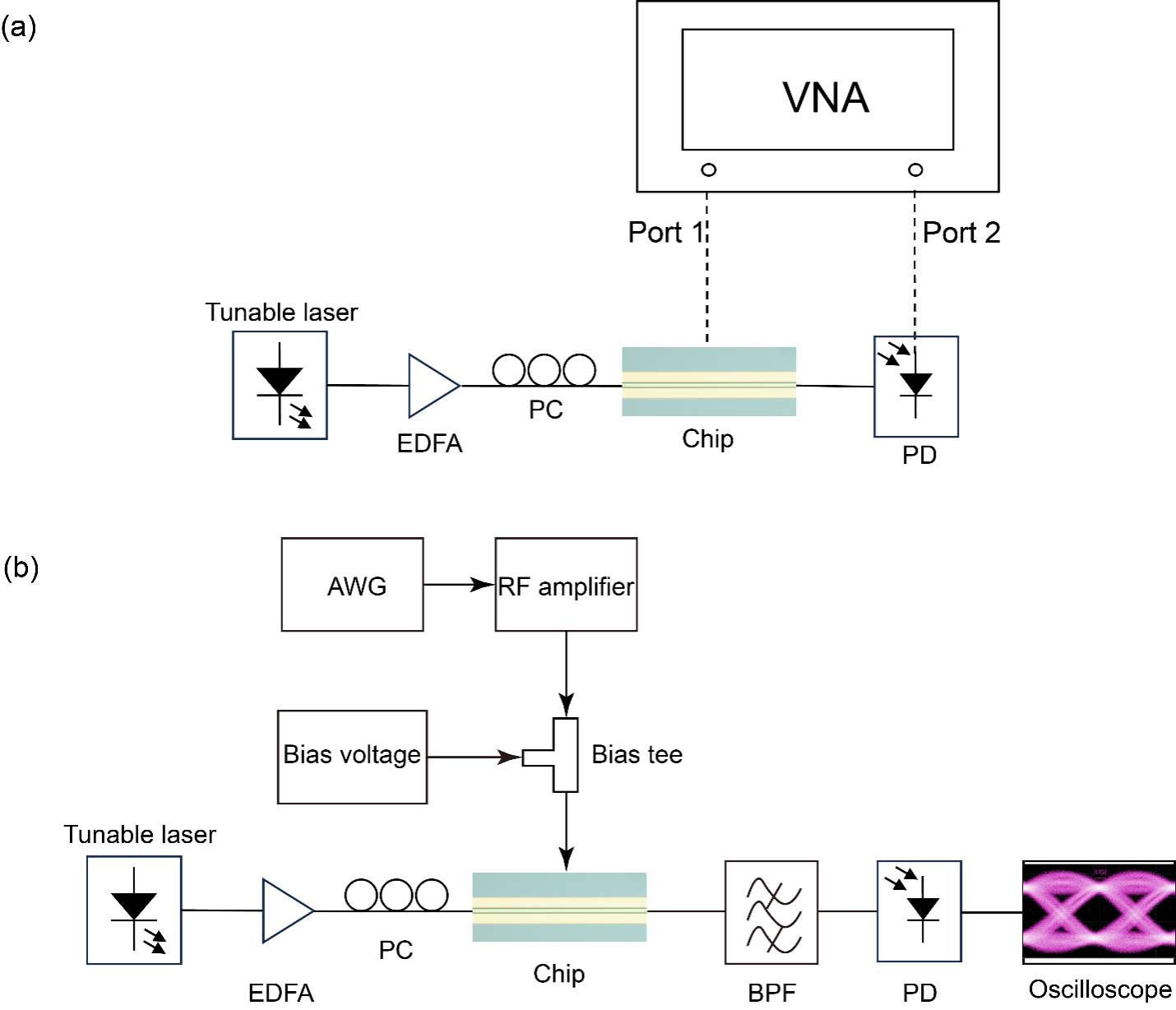


**Figure S7.** Experimental characterization setup. (a) Experimental setup for modulation bandwidth characterization. (b) Experimental setup for eye diagrams characterization. PC polarization controller, PD photodetector, EDFA Erbium doped optical fiber amplifier, BPF bandpass optical filter.
